# miR-933 accelerates the growth of liver cancer cells by enhancing pyruvate kinase isoform M2

**DOI:** 10.1016/j.gendis.2023.101186

**Published:** 2023-11-30

**Authors:** Liyan Wang, Shujie Li, Xinlei Liu, Sijie Xie, Shuting Song, Xiaoxue Jiang, Dongdong Lu

**Affiliations:** Shanghai Putuo People's Hospital, School of Life Science and Technology, Tongji University, Shanghai 200092, China

MicroRNAs (miRNAs) have been found to play an important role in human tumorigenesis. A study indicates that the plasma level of miR-933 was elevated in patients with dementia.^1^ Notably, miR-933 (RS79402775) may contribute to the reduction of gastric cancer susceptibility.^2^ Moreover, the study found that the miR-933 expression level in superficial diffuse melanoma was lower than in nodular melanoma.^3^ Furthermore, miR-933 has become a reliable prognostic tool for tumor progression.^4^ Strikingly, single nucleotide polymorphisms of miR-933 were found to be associated with the risk of papillary thyroid cancer.^5^ In this study, we demonstrate that miR-933 accelerates the growth of liver cancer cells by enhancing the expression of pyruvate kinase isoform M2 (*PKM2*) and increasing DNA damage repair. These results provide a basis for research on liver cancer prevention.

To address the effect of miR-933 on human liver cancer cells, the human liver cancer cell Hep3B was infected with rLV and rLV-miR-933 (Fig. S1A). miR-933 was overexpressed in the rLV-miR-933 group in contrast with the rLV group (Fig. 1A; Fig. S1B). The proliferation ability (*P* < 0.01) (Fig. S1C) and colony formation ability (9.59 % ± 2.96 % *vs*. 52.48 % ± 3.05 %, *P* = 0.00066) (Fig. S1D a, b) were significantly increased in the rLV-miR-933 group in contrast with the rLV group. As shown in Figure 1B a, b and Figure S1E–G, the weight and the poor differentiation degree of transplanted tumors were significantly increased in the rLV-miR-933 group (0.377 ± 0.032 g *vs*. 0.908 ± 0.067 g, *p* = 0.0000014). Furthermore, mature miR-933 was significantly decreased in the rLV-Cas9-miR-933(1) group and rLV-Cas9-miR-933(2) group in contrast with the rLV-Cas9 group (Fig. 1C). The growth ability *in vitro* and *in vivo* was decreased in the rLV-Cas9-miR-933(1) group and rLV-Cas9-miR-933(2) group in contrast with the rLV-Cas9 group (Fig. 1D a, b; Fig. S2A–E).

**Figure 1:**
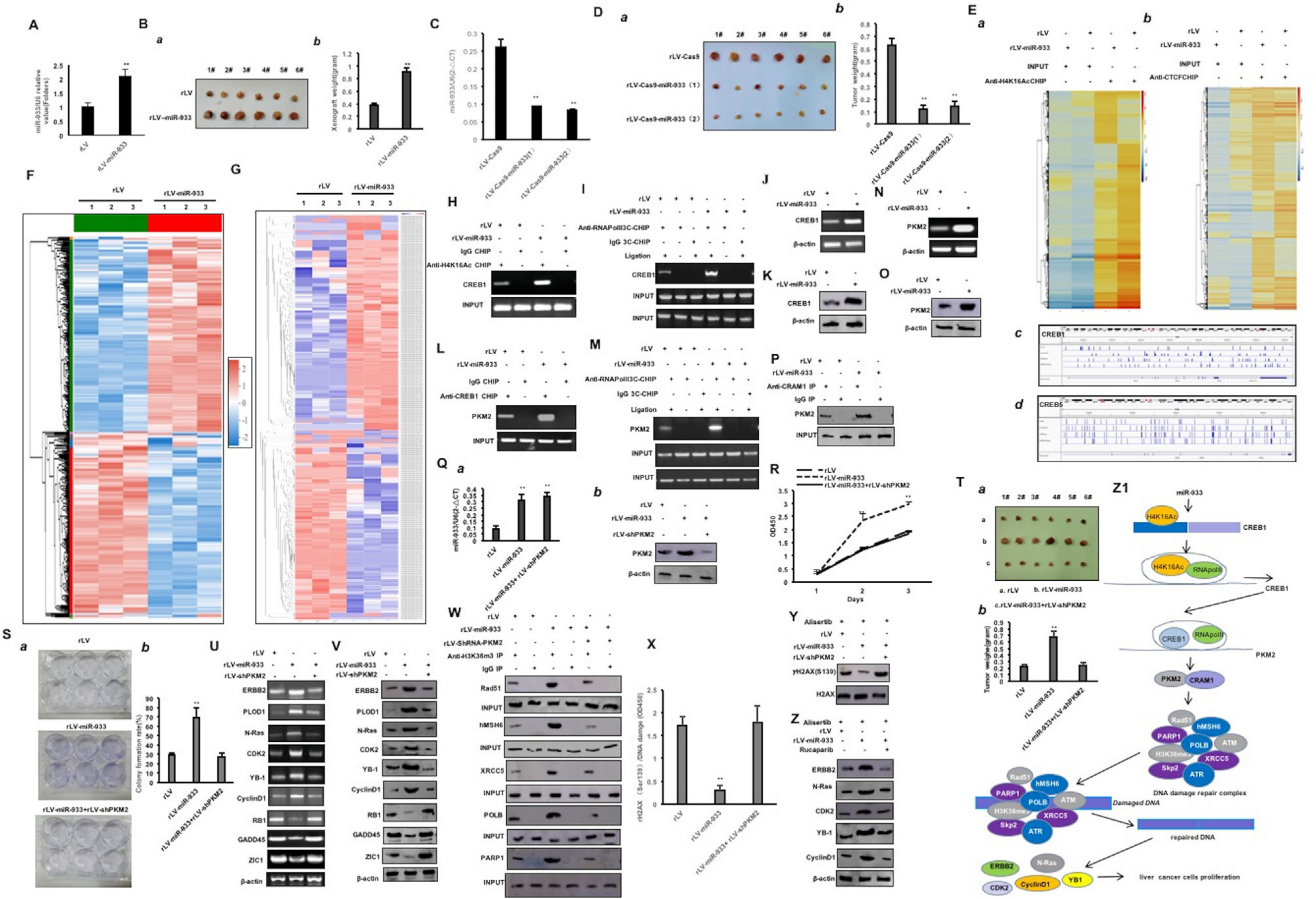
miR-933 accelerates the growth of liver cancer cells by enhancing *PKM2*. **(A)** Quantitative reverse-transcription (RT)-PCR was used to detect the mature miR-933. **(B)** The xenograft tumor was dissected (*a*) and the tumor size (g) was put into comparison between groups (*b*). **(C)** Hep3B cells infected with rLV-Cas9-miR-933 and miR-933 were detected by real-time RT-PCR. CCK8 method was used to determine the cell proliferation ability. **(D)** The xenograft tumor was dissected (*a*) and the tumor size (g) was put into comparison between groups (*b*). **(E)** Chromatin immunoprecipitation sequencing (CHIP-Seq) with anti-H4K16Ac (*a*) and the hierarchical clustering analysis (*b*); CHIP-Seq with anti-CTCF (*c*) and the hierarchical clustering analysis (*d*). **(F)** miR-933 affects the transcriptome as shown in the heat map analysis (cluster). **(G)** miR-933 alters proteomics as shown in the differential protein cluster heatmap. **(H)** CHIP analysis was performed with anti-H4K16Ac. The PCR amplification was carried out using primers designed according to the DNA of the CREB1 promoter region. **(I)** 3C-CHIP analysis was performed with anti-RNAPolII. **(J)** The transcriptional ability of CREB1 was detected by RT-PCR. **(K)** The translational ability of CREB1 was detected by western blotting. **(L)** CHIP analysis was performed with anti-CREB1. The PCR amplification was carried out using primers designed according to the DNA of the PMK2 promoter region. **(M)** 3C-CHIP analysis was performed with anti-RNAPolII. **(N)** PKM2 was detected by RT-PCR. **(O)** PKM2 was detected by western blotting. **(P)** Co-immunoprecipitation analysis with anti-CARM1 and western blot analysis with anti-PKM2. **(Q)** PKM2 was detected with western blotting (*a*) and the quantitative RT-PCR was performed to detect the mature miR-933 (*b*). **(R)** CCK8 method was used to determine the cell proliferation ability. **(S)** The colony formation ability. (*a*) Photos of plate colonies. (*b*) Colony forming rate (%). **(T)** The xenograft tumor was dissected (*a*) and the tumor size (g) was put into comparison between groups (*b*). **(U)** RT-PCR analysis for ERBB2, POLD1, N-Ras, CDK2, YB1, CyclinD1, RB1, GADD45, and ZIC1. **(V)** Western blot analysis for ERBB2, POLD1, N-Ras, CDK2, YB1, CyclinD1, RB1, GADD45, and ZIC1. **(W)** Immunoprecipitation analysis with anti-H3K36me3, anti-Rad51, anti-hMSH6, anti-XRCC6, anti-POLB, and anti-PARP1. **(X)** γH2AX(S139) detection. **(Y)** Western blot analysis with anti-γH2AX(S139). **(Z)** Western blot analysis with anti-ERBB2, anti-N-Ras, anti-CDK2, anti-CyclinD1, and anti-YB1. **(Z1**) The schematic diagram. miR-933 accelerates the growth of liver cancer cells and increases the binding ability of H4K16Ac and RNAPolII to the promoter region of *CREB1*. Thereby, the expression of *CREB1* was increased. Next, miR-933 increases the binding ability of CREB1 and RNApolII to the promoter region of *PKM2*, and the expression of *PKM2* was increased. Therefore, miR-933 increases the interaction between PKM2 and CARM1. Then, miR-933 enhances the complex formation of H3K36me3-Rad51-hMSH6-XRCC6-POLB-PARP1 dependent on PKM2-CARM1 and then increases the DNA damage repair ability, which enhances the expression of ERBB2, N-Ras, CDK2, CyclinD1, and YB1. Ultimately, miR-933 accelerates the growth of liver cancer cells by enhancing *PKM2* expression and DNA damage repair.

To explore how miR-933 affects the epigenetic regulation of some genes, chromatin immunoprecipitation sequencing with anti-H4K16Ac or anti-CTCF was performed. As shown in Figure 1E a–d, Figure S3A–J, and Figure S4A–J, there was a difference in the modification distribution of H4K16Ac on the promoter region of some genes, such as *PRKAR2B*, *SMAD5*, *CARM1*, *CREB5*, *CDK1*, *CDK7*, *CDK19*, *CTCF*, *GABARAPL2*, *CDK14*, *CREB1*, *PKM2*, *Rad51B*, *HDAC3*, *MAP2K3*, *PARP2*, *POLR3B*, *Rad51*, *Rad51B*, *SAMD4A*, and *PPP2R2C*, and a difference in the modification distribution of CTCF on the promoter region of some genes, such as *HAT1*, *KAT6A*, *CARM1*, *MAP3K9*, *CREB1*, *PKM2*, *CREB5*, *RAB11A*, *CTCF*, *Rad51*, *HDAC8*, and *HDAC9* between the rLV group and rLV-miR-933 group. Besides, there was a difference in peak binding motif in the genomic region between the rLV group and rLV-miR-933 group, such as CTCTCTCT, TCTCTCTC, GTGTGTGT, TGTCTGTC, CACACACA, TGGTGGTG, and TACTCTAC (Fig. S3J). To study the effect of miR-933 on the transcriptome in human liver cancer, the total RNA was extracted for RNA sequencing. As shown in Figure 1F and Figure S5A–G, these results showed that the expression of 170 genes was up-regulated and the expression of 162 genes was down-regulated between the rLV group and rLV-miR-933 group. The up-regulated genes mainly include *ABHD6*, *ADD2*, *AFTPH*, *ANTXR2*, *ARHGAP22*, *BICD1*, *CNOT3*, *CPEB4*, *EGR1*, *ERG3*, *ERCC2*, *FBXO30*, *H2BC13*, *JUNB*, *MARK4*, *MLLT11*, *MMP9*, *PIK3C2A*, *PDGFA*, *PRKAR2B*, *RAB7A*, *SUZ12*, *TOP2B*, *ZNF35*, *N-Ras*, *PCNA*, *C-FOS*, *C-myc*, *CDK2*, and *CyclinE*. The down-regulated genes mainly include *ADAM19*, *ADGRB2*, *BCAR3*, *ATG4B*, *CDC16*, *CHAC1*, *CTNNA3*, *COPS7B*, *DCN*, *EPN2*, *DLC1*, *H2AZ1*, *TWIST2*, *UBE3A*, *SEC16B*, *RPL34*, *RBL1*, *POLB*, *OLFM1*, *OLFML3*, *MAT2A*, *MFHAS1*, *HSPA1A*, *HOXC8*, *DGR2*, *MANF*, *ZNF367*, *GAN*, *STK17B*, *CLASRP*, *P73*, *PTEN*, *ZIC1*, and *P18*. The up-regulated KEGG pathways mainly include the Ras signaling pathway, MAPK signaling pathway, JAK-STAT signaling pathway, and cGMP-PKG signaling pathway. Furthermore**,** to study the effect of miR-933 on proteomics in human liver cancer, the total protein was extracted and then the proteolytic peptides were analyzed by label-free mass spectrometry. As shown in Figure 1G and Figure S6A–G, the results showed that the expression of 283 proteins was up-regulated and the expression of 324 proteins was down-regulated between the rLV group and rLV-miR-933 group. The up-regulated proteins include ADD1, ANXA4, ATP6V1H, ATG3, BCLAF1, CBX1, EHD3, CDK4, DDX54, XPNPEP1, EPHX1, FAR1, GLIPR2, H1-10, ISOC1, MAPK3, PKM, RAB21, RRAS, TNRC5, TOMM40, TOMM34, S100SA4, RAB7A, PKM2, LUC7L2, UPF3B, H3K27me3, ABCE1, HP1a, CDK4, PCNA, METTL3, SUV39h1, Pim1, N-Ras, MYB, C-FOS, C-myc, CDK2, and CyclinE. The down-regulated proteins include ACON, LRRFIP1, CBX5, CISD3, CRK, CTSZ, DNAJC7, DNMT1, H2AZ2, H10, HAT1, HYPK, WDR12, YARS2, UAP1L1, TOP2A, SNX6, SAR1A, RRP9, PTMAP7, POLE3, NDUFA6, MYDGF, MANF, NFkB, BECN1, TMEN111, EZH2, P73, PTEN, ZIC1, and P18. The up-regulated KEGG pathways mainly include the Rap1 signaling pathway, glycolysis, and Ras signaling pathway. Importantly, it is worth mentioning that miR-933 enhances the expression of *PKM2*.

To further investigate how miR-933 affects the expression of *PKM2* and the interaction between PKM2 and CARM1 in human liver cancer, we explored whether several important genes were involved in the regulation of this function. First, the binding ability of H4K16Ac to the promoter region of *CREB1* was increased in the rLV-miR-933 group in contrast with the rLV group (Fig. 1H). The binding ability of RNAPolII to the promoter-enhancer loop of *CREB1* was increased in the rLV-miR-933 group in contrast with the rLV group (Fig. 1I). Therefore, the transcriptional ability of *CREB1* was increased in the rLV-miR-933 group in contrast with the rLV group (Fig. 1J). The expression of *CREB1* was increased in the rLV-miR-933 group in contrast with the rLV group (Fig. 1K). Next, the binding ability of CREB1 to the promoter region of *PKM2* was increased in the rLV-miR-933 group in contrast with the rLV group (Fig. 1L). Moreover, the binding ability of RNAPolII to the promoter-enhancer loop of *PKM2* was increased in the rLV-miR-933 group in contrast with the rLV group (Fig. 1M). The binding ability of RNAPolII to the *PKM2* promoter probe was increased in the rLV-miR-933 group in contrast with the rLV group (Fig. S7A). The transcriptional activity of *PKM2* was increased in the rLV-miR-933 group in contrast with the rLV group (Fig. S7B). The transcriptional ability of *PKM2* was increased in the rLV-miR-933 group in contrast with the rLV group (Fig. 1M). The expression of PKM2 was increased in the rLV-miR-933 group in contrast with the rLV group (Fig. 1O). Ultimately, the interaction between PKM2 and CARM1 was increased in the rLV-miR-933 group in contrast with the rLV group (Fig. 1P). Collectively, these observations suggest that miR-933 could enhance the expression of PKM2 via *CREB1* and the interaction between PKM2 and CARM1 in *human* liver cancer.

Given miR-933's role in enhancing the expression of *PKM2* and the interaction between PKM2 and CARM1, we considered whether miR-933 resulted in carcinogenic functions dependent on *PKM2* in liver cancer. First, *PKM2* expression was increased in the rLV-miR-933 group and decreased in the rLV-miR-933+rLV-ShRNA-PKM2 group in contrast with the rLV group, respectively (Fig. 1Q *a*). miR-933 was increased in the rLV-miR-933 group and rLV-miR-933+rLV-ShRNA-PKM2 group in contrast with the rLV group (Fig. 1Q *b*). Although the proliferation ability was increased in rLV-miR-933 group in contrast with the rLV group (24 h: *P* = 0.0024; 48 h: *P* = 0.0088), it was not changed in the rLV-miR-933+rLV-ShRNA-PKM2 group in contrast with the rLV group, respectively (24 h: *P* = 0.00996; 48 h: *P* = 0.148) (Fig. 1R). Although the colony formation ability was increased in the rLV-miR-933 group in contrast with the rLV group (28.82 % ± 2.69 % *vs*. 69.06 % ± 11.14 %, *P* = 0.00017), it was not changed in the rLV-miR-933+rLV-ShRNA-PKM2 group in contrast with the rLV group (28.82 % ± 2.69 % *vs*. 27.17 % ± 4.11 %, *P* = 0.094) (Fig. 1S *a*, *b*). Although the weight of transplanted tumors was increased in the rLV-miR-933 group in contrast with the rLV group (0.218 ± 0.029 g *vs*. 0.675 ± 0.091 g, *P* = 0.0000057), it was not changed in the rLV-miR-933+rLV-ShRNA-PKM2 group in contrast with rLV group (0.218 ± 0.029g *vs*. 0.237 ± 0.054 g, *P* = 0.258) (Fig. 1T *a*, *b*). Although the appearance time of xenograft tumors was decreased in the rLV-miR-933 group in contrast with the rLV group (9.5 ± 1.05 days *vs*. 7.17 ± 0.75 days, *P* = 0.0043), it was not changed in the rLV-miR-933+rLV-ShRNA-PKM2 group in contrast with the rLV group (9.5 ± 1.05 days *vs*. 9.83 ± 1.72days, *P* = 0.319) (Fig. S8A). As shown in Figure S8B, although the well-differentiated cells were decreased and the poorly differentiated cells were increased in the rLV-miR-933 group in contrast to the rLV group, respectively, it was not significantly changed in the rLV-miR-933+rLV-ShRNA-PKM2 group in contrast with the rLV group. Although the expression of PCNA was increased in the rLV-miR-933 group in contrast with the rLV group (35.39 % ± 4.22 % *vs*. 74.01 % ± 11.76 %, *P* = 0.000247), it was not changed in the rLV-miR-933+rLV-ShRNA-PKM2 group in contrast with the rLV group (35.39 % ± 4.22 % *vs*. 33.54 % ± 7.11 %, *P* = 0.34 (Fig. S8C). Although the expression of *ERBB2*, *POLD1*, *N-Ras*, *CDK2*, *YB1*, and *CyclinD1* was increased in the rLV-miR-933 group and the expression of *RB1*, *GADD45*, and *ZIC1* was decreased in the rLV-miR-933 group in contrast to rLV group, it was not altered in rLV-miR-933+rLV-shRNA-PKM2 group in contrast with rLV group (Fig. 1U, V). Although the interactions between H3K36me3 and Rad51, hMSH6, XRCC6, POLB, and PARP1 were increased in the rLV-miR-933 group in contrast with the rLV group, respectively, it was not changed in the rLV-miR-933+rLV-ShRNA-PKM2 group in contrast with rLV group (Fig. 1W). Although the DNA damage marker γH2AX(S139) was significantly decreased in the rLV-miR-933 group in contrast with the rLV group, it was not changed in the rLV-miR-933+rLV-ShRNA-PKM2 group in contrast with rLV group, respectively (Fig. 1X, Y). Although the expression of ERBB2, N-Ras, CDK2, CyclinD1, and YB1 was increased in the rLV-miR-933 group in contrast with the rLV group, it was not changed in the rLV-miR-933+Rucaparib (a DNA damage repair inhibitor) group in contrast with the rLV group (Fig. 1Z). Collectively, these observations suggest that PKM2 regulates the carcinogenic functions of miR-933 in liver cancer.

Obviously, our findings are noteworthy that miR-933 affects epigenetic regulation, transcriptome, proteome, and several signaling pathways. In particular, our results suggest that miR-933 enhances the expression of PKM2 and the interaction between PKM2 and CARM1 in liver cancer. To date, we demonstrate that miR-933 accelerates the growth of liver cancer cells by enhancing PKM2 expression and DNA damage repair. That is, miR-933 increases the binding ability of H4K16Ac and RNAPolII to the promoter region of *CREB1*. Thus, the expression of *CREB1* is increased in liver cancer cells. Next, miR-933 increases the binding ability of CREB1 and RNApolII to the promoter region of *PKM2*, and the expression of *PKM2* is increased. Therefore, miR-933 increases the interaction between PKM2 and CARM1. Then, miR-933 enhances the complex formation of H3K36me3-Rad51-hMSH6-XRCC6-POLB-PARP1 dependent on PKM2-CARM1and then increases the DNA damage repair ability, which enhances the expression of ERBB2, N-Ras, CDK2, CyclinD1, and YB1 (Fig. 1Z1). This first discovery provides a basis for the prevention and treatment of human liver cancer.

## Ethics declaration

All methods were carried out in accordance with the approved guidelines. All experimental protocols were approved by a Tongji University Institutional Committee. The study was reviewed and approved by the China National Institutional Animal Care and Use Committee (animal ethical number: TJAB04220101).

## Author contributions

Dongdong Lu conceived the study and participated in the study design, performance, coordination, and manuscript writing. Liyan Wang, Shujie Li, Xinlei Liu, Sijie Xie, Shuting Song, and Xiaoxue Jiang performed the research. All authors read and approved the final manuscript.

## Funding

This study was supported by grants from the National Natural Science Foundation of China (No. 82073130) and the Science and Technology Commission of Shanghai Municipality Shanghai Science and Technology Plan Basic Research Field Project (China) (No. 20JC1411400).

## Conflict of interests

The authors declare that they have no competing interests.
