# Supplementary material for: miR-933 accelerates the growth of liver cancer cells by enhancing pyruvate kinase isoform M2
Source: Genes Dis. 2023 Nov 30;11(4):101186. doi: 10.1016/j.gendis.2023.101186 (PMC10909591; doi:10.1016/j.gendis.2023.101186)
Supplement: Multimedia component 1 [file mmc1.docx]

**Supplemental Materials and Methods**

**Cell Lines, Lentivirus** Human liver cancer cell line (Hep3B) was maintained in Dulbecco’s modified Eagle medium (Gibco BRL Life Technologies) in a humidified atmosphere of 5% CO_2_ incubator at 37ºC. rLV, rLV-miR-933 were purchased from Wu Han viraltherapy Technologies Co. Ltd. pGFP-V-RS was purchased from Origene (Rockville, MD,USA).

**Western blotting** Total proteins were separated on a 10% SDS-PAGE and transferred onto nitrocellulose membrane. The blots were incubated with antibody(1:1000-2000) at 4°C overnight. Following three washes, membranes were then incubated with secondary antibody(1:2000) at 4°C overnight. Signals were visualized by ECL system.

**Chromatin immunoprecipitation (CHIP) assay** Crossed-linked cells were washed with phosphate-buffered saline, resuspended in lysis buffer, and sonicated. Chromatin extracts were pre-cleared with Protein-A/G-Sepharose beads, and immunoprecipitated with specific antibody on Protein-A/G-Sepharose beads. After washing, elution and de-cross-linking, the ChIP DNA was amplified and identified.

### Cellular proliferation Assay The cell proliferation abilty was measured using CCK8 kit according to the manufacturer instruction(Beyotime Biotechnology).

**Tumorigenesis test *in vivo*** Four-weeks male athymic Balb/C mice were injected with Hep3B at the armpit area subcutaneously. The mice were observed over 4 weeks, and then sacrificed to recover the tumors. The use of mice for this work was reviewed and approved by the institutional animal care and use committee in accordance with China national institutes of health guidelines.

**Chip-Seq CHIP** sequencing analysis was performed according to according to the manufacturer operation manual (Novogene Co., Ltd., Beijing Nuohe Zhiyuan Technology Co., Ltd.)

**RNA sequencing analysis** RNA sequencing analysis was performed according to according to the manufacturer operation manual (Shanghai Majorbio Bio-pharm Technology Co.,Ltd)

**Mass spectrometric analysis** Mass spectrometric analysis of enzyme hydrolyzed peptides of protein without label free was performed according to according to the manufacturer operation manual (Shanghai Majorbio Bio-pharm Technology Co.,Ltd)

**Immunoprecipitation protein mass spectrometry** Immunoprecipitation protein mass spectrometry analysis was performed according to the manufacturer operation manual(Shanghai Majorbio Bio-pharm Technology Co.,Ltd).

**Supplemental Figure & Figure Legends**

**Fig.S1**

**
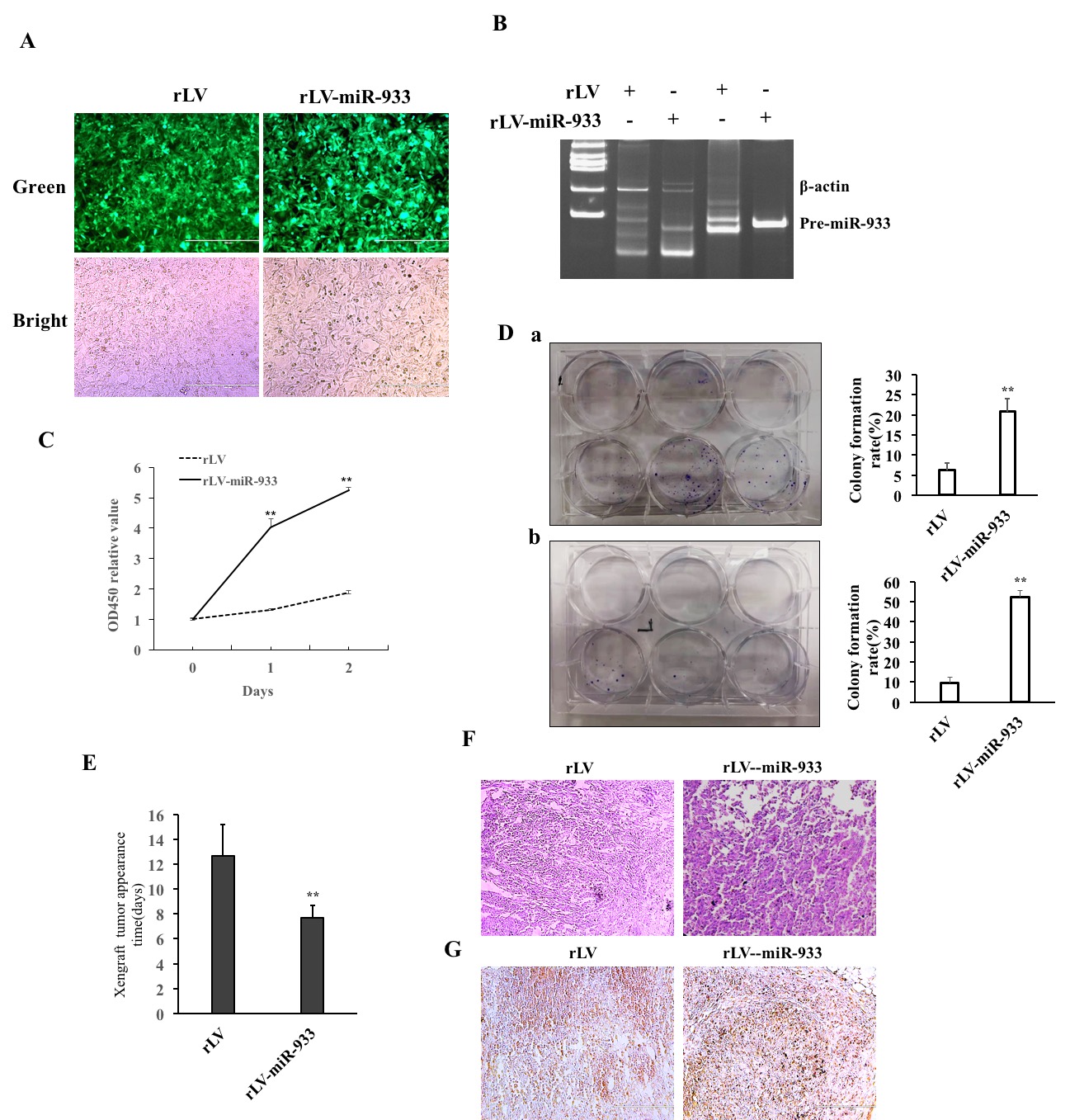
**

**FigureS1** miR-933 promotes the growth of liver cancer cells. A. Hep3B cells were infected with rLV-miR-933 and the pictures were taken under fluorescence microscope. B. The precursor of miR-933 was detected by RT-PCR. C. CCK8 method was used to determine the cell proliferation ability. D. The colony forming ability of cells was measured. a. photos of plate colonies. b. Colony forming rate(%). E. Comparison of tumor appearance time(days). F. Hematoxylin eosin (HE) (original registration ×100). G.anti-PCNA immunohistochemical staining (original registration×100). .

**Fig.S2**


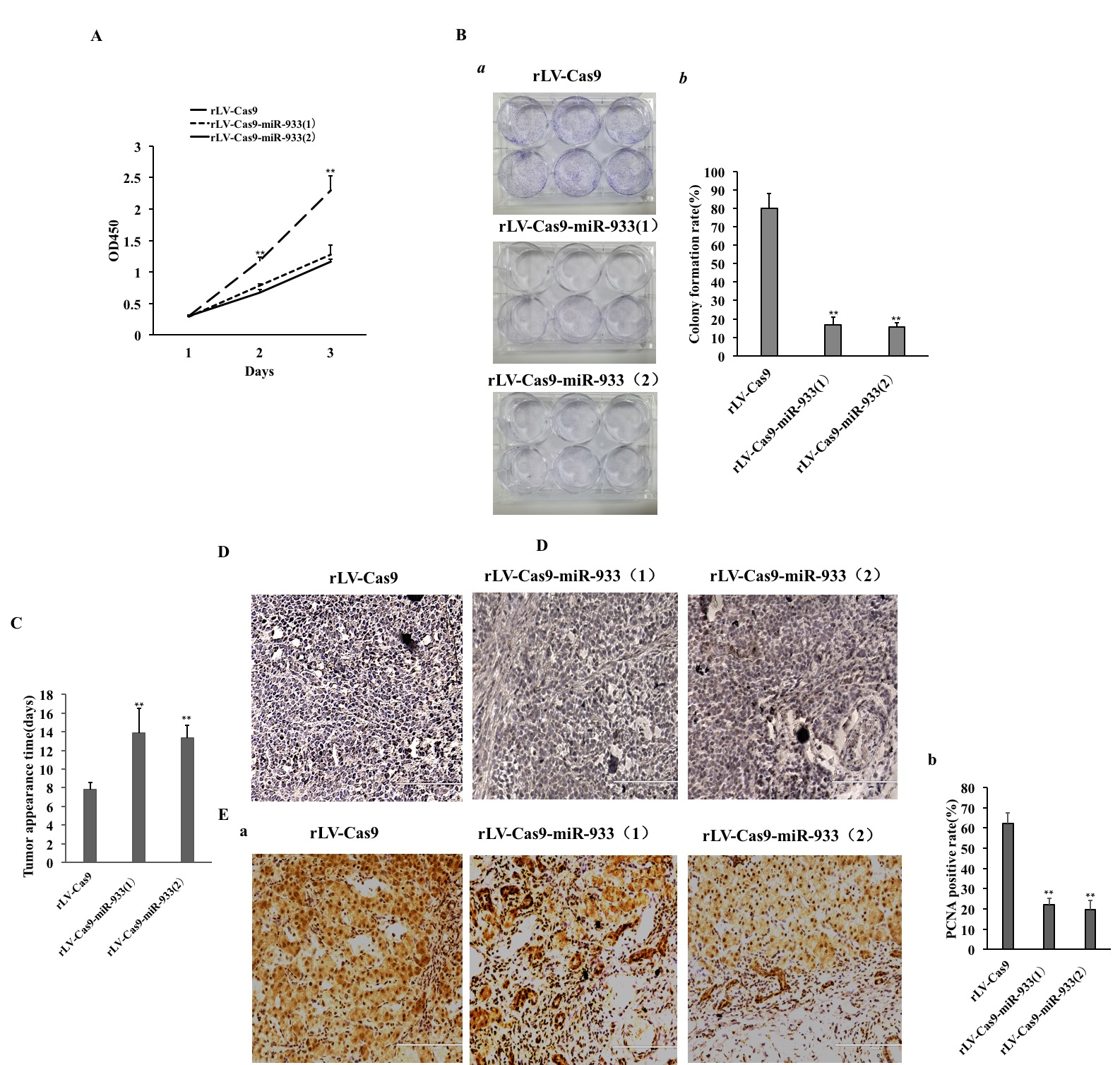


**FigureS2** miR-933 knockdown inhibits the growth of liver cancer cells. rLV-cas9-miR-933 was prepared with pLVX-U6-miR-933sgRNA-Cas9-ZsGreen.

miR-933 sgRNA sequence：5’-TCGAGTCAGCCGTGTGCGCAGGG-3’. The ACCGGTCGAGTCAGCCGTGTGCGCAGTTTTAGAGCTAGAAATAGCAAGTTAAAATAAGGCTAGTCCGTTATCAACTTGAAAAAGTGGCACCGAGTCGGTGCTTTTTTGAATTC-3’ was cloned into pLVX-U6-CMV-Cas9-P2A-ZsGreen**(**AgeI+EcoRI). A. CCK8 method was used to determine the cell proliferation ability. B. The colony forming ability of cells was measured. a. photos of plate colonies. b. colony formation rate. C. Comparison of tumor appearance time(days). D. Hematoxylin eosin (HE) staining (original registration×100). E. anti-PCNA immunohistochemical staining. a. anti-PCNA immunohistochemical staining picture(original registration×100).b.PCNA positive rate(%).

**Fig.S3**

**
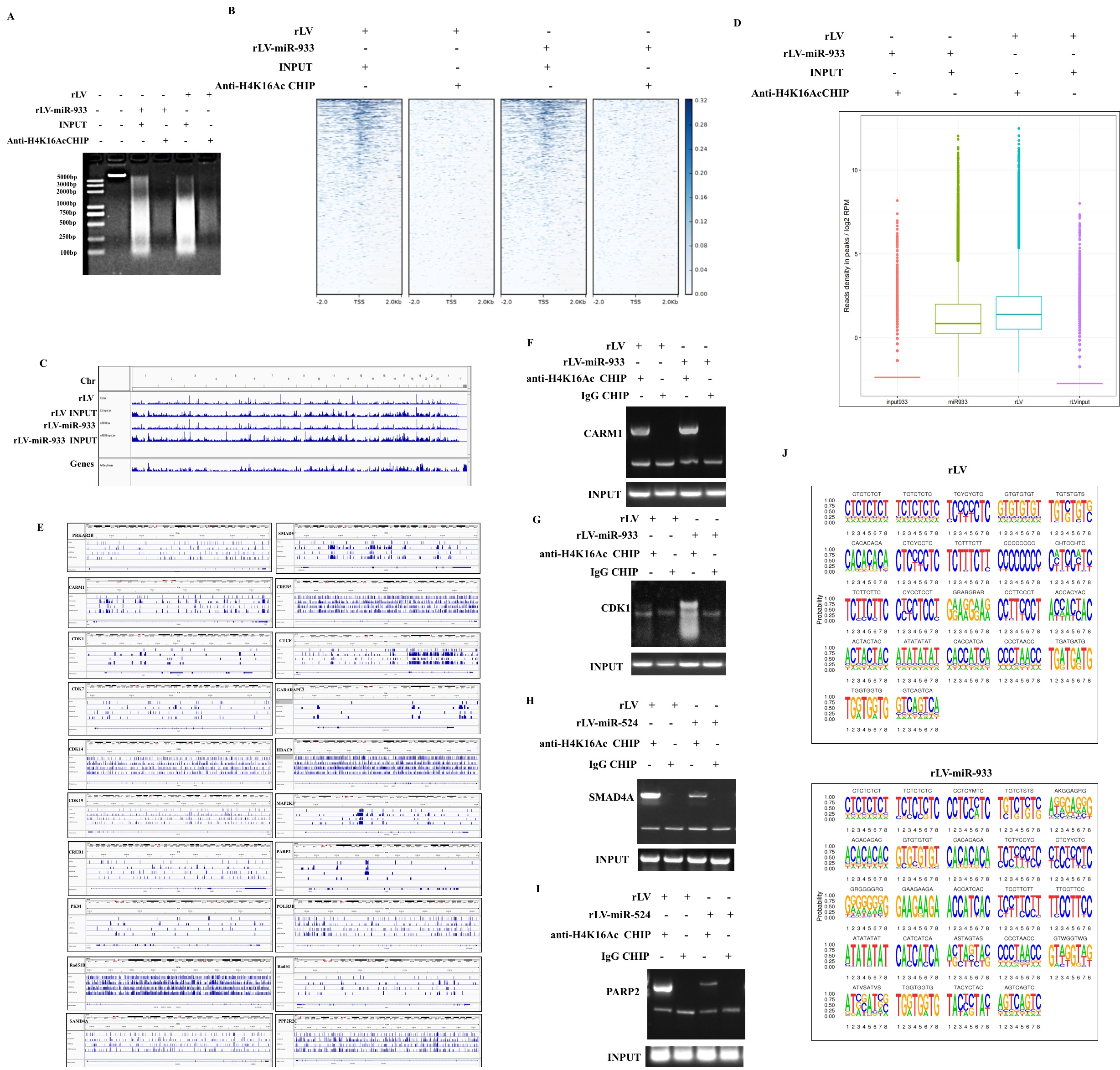
**

**FigureS3** Chromatin immunoprecipitation sequencing (Chip-Seq) with anti-H4K16Ac. A. Cells were cross-linked by formaldehyde of 1% and DNA was extracted. Then the DNA fragments after ultrasonic fragmentation were identified by 1% agarose gel electrophoresis. B. The average signal distribution on the 2KB region upstream and downstream of TSS. C. IGV browser interface (Demo). D. Boxplot plot of reads density distribution in peak region (RPM). E. IGV browser interface (Demo). F-I. anti-H4K16AcCHIP. J. The peak binding motif in the genomic region between rLV group and rLV-miR-933 group.

**Fig.S4**

**
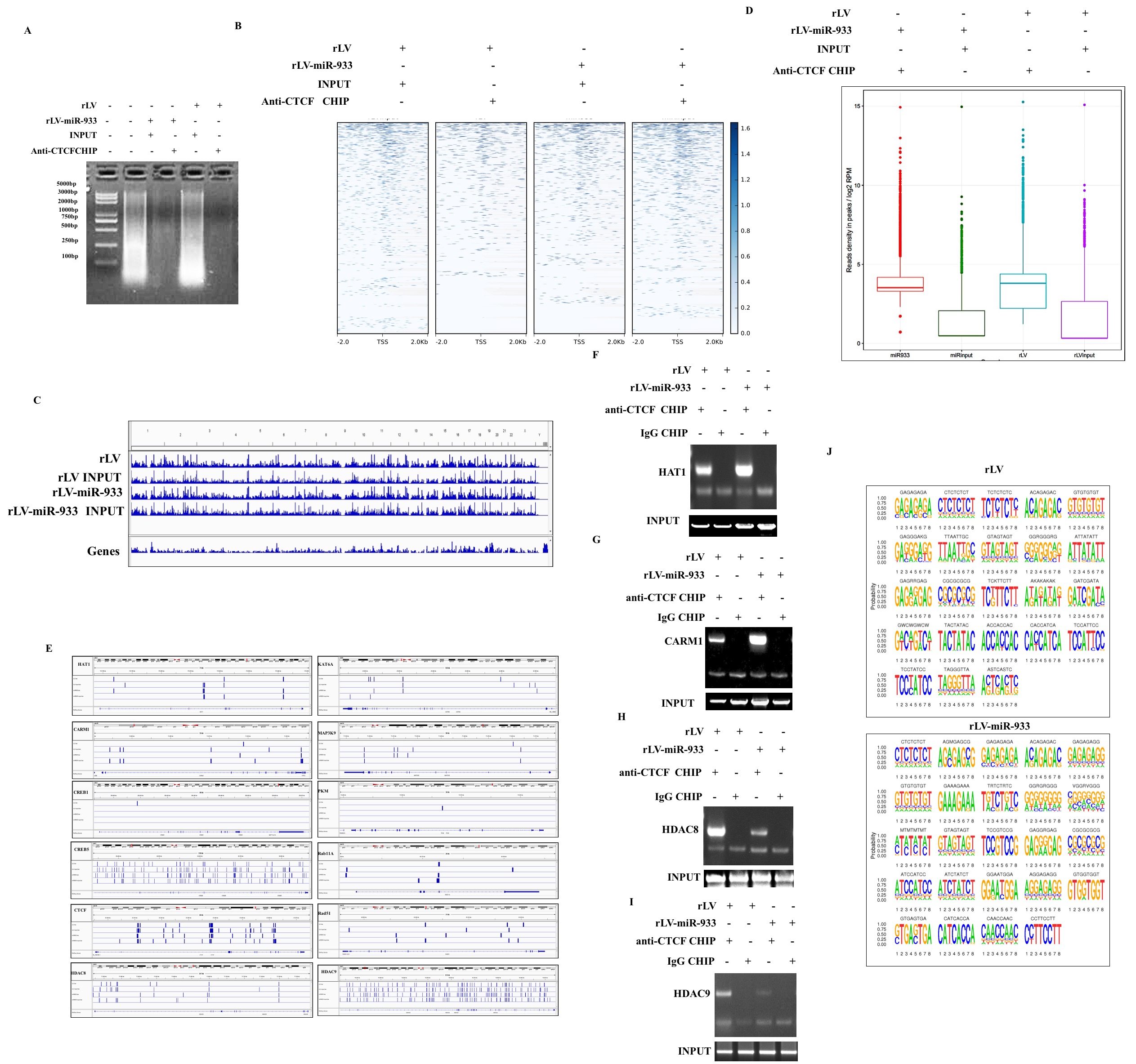
**

**FigureS4** Chromatin immunoprecipitation sequencing (Chip-Seq) with anti-CTCF. A. Cells were cross-linked by 1% formaldehyde and DNA was extracted. Then the DNA fragments after ultrasonic fragmentation were identified by 1% agarose gel electrophoresis. B.The average signal distribution on the 2KB region upstream and downstream of TSS. C. IGV browser interface (Demo). D. Boxplot plot of reads density distribution in peak region (RPM). E. IGV browser interface (Demo).F-I.anti-CTCF CHIP. J. The peak binding motif in the genomic region between rLV group and rLV-miR-933 group.

**Fig.S5**

**
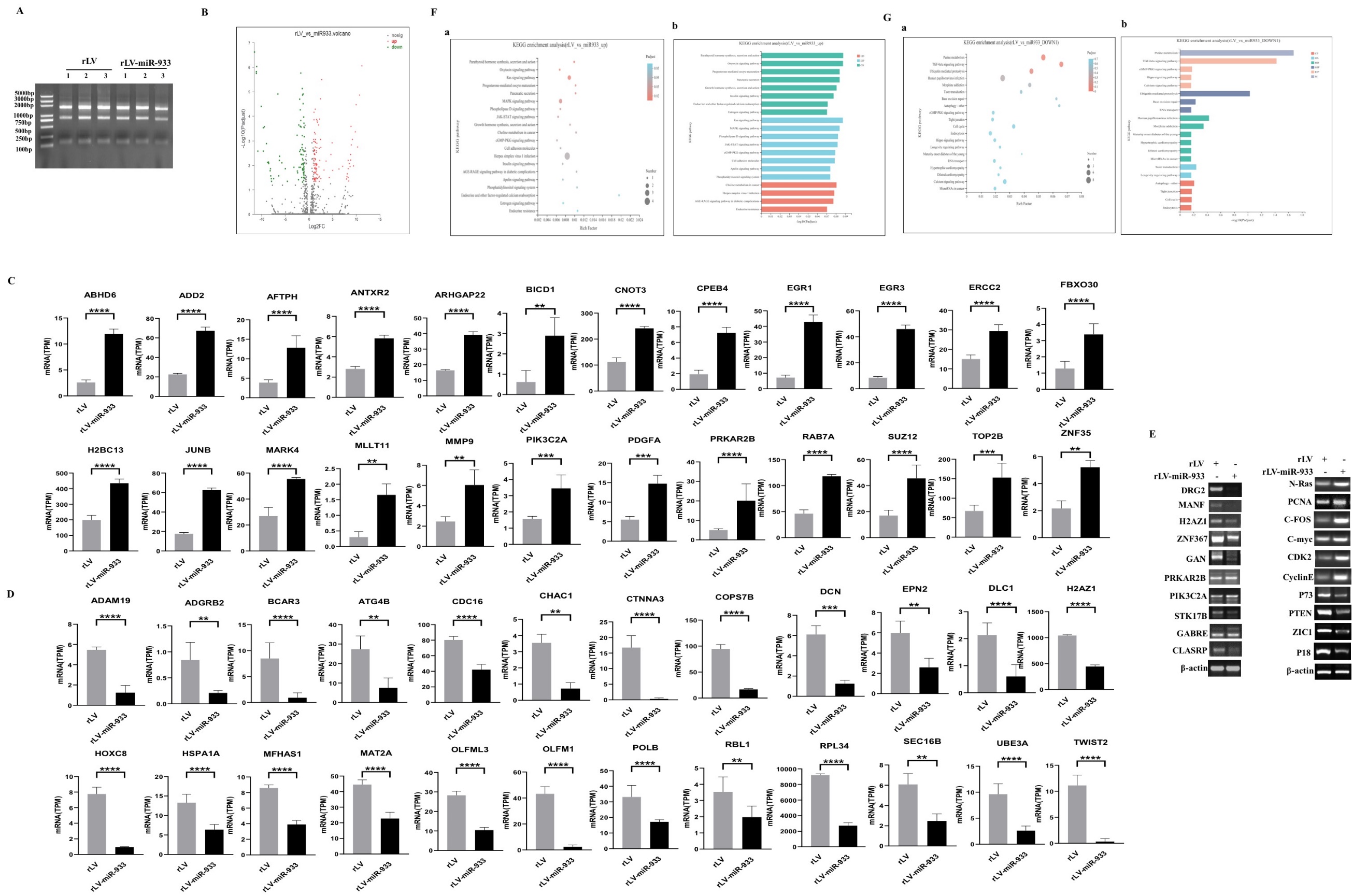
**

**Figure5** miR-933 affects on the transcriptome. A. Total RNA was extracted and detected by 1% agarose gel electrophoresis. B. The volcanic map. C. Up-regulated genes. D. Down-regulated genes. E. RT-PCR analysis. β-actin as intenal control. F. Up-regulated KEGG enrichment analysis. a.scatter diagram. b. Histogram. G. downregulated KEGG enrichment analysis ( scatter diagram).a. scatter diagram. b. Histogram.

**Fig.S6**


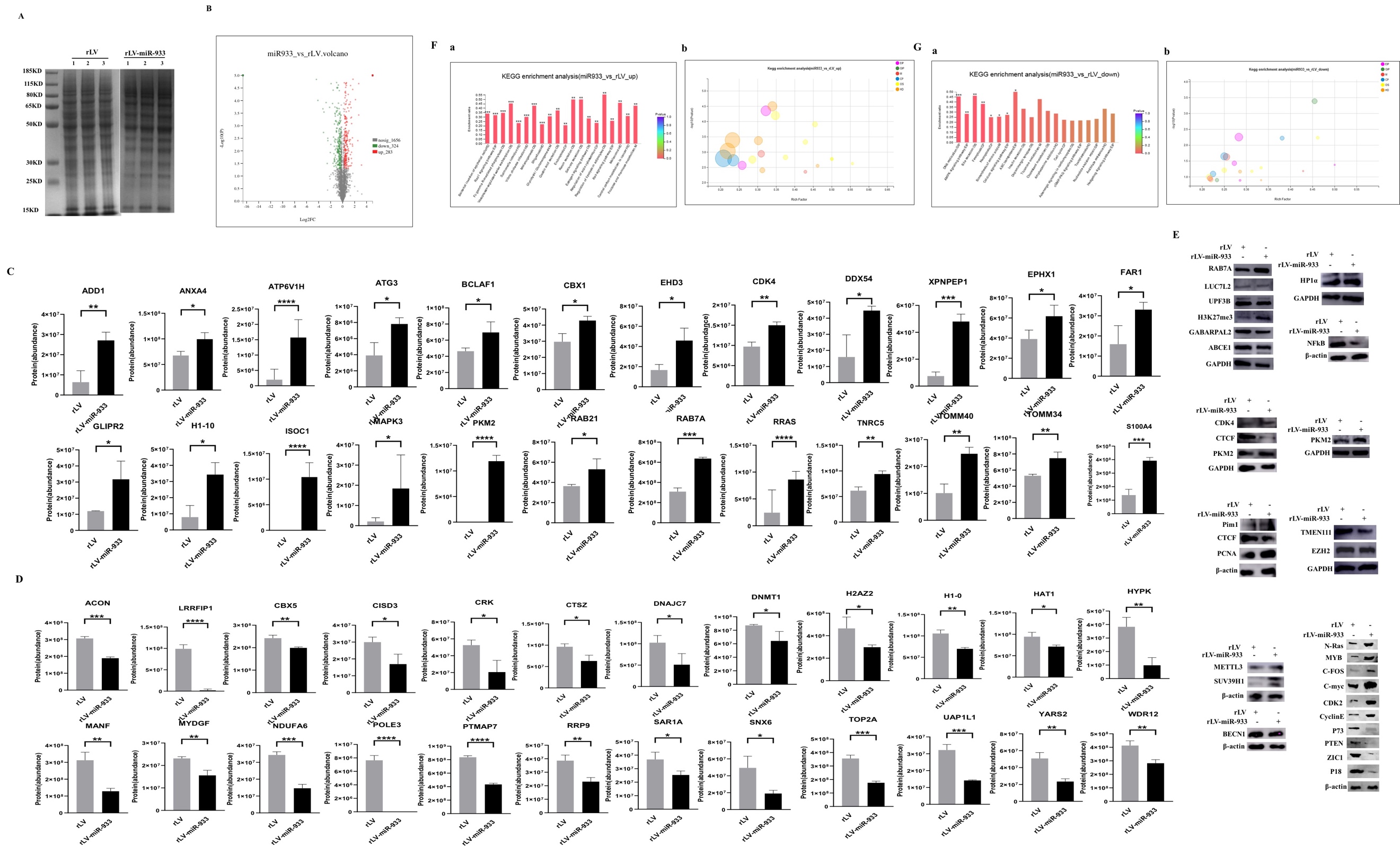


**FigureS6** miR-933 alters proteomics. A. The total protein was extracted and analyzed by 10% SDS-PAGE electrophoresis. B. Differential protein volcano map . C. Histogram of up-regulated proteins . D. Histogram of down-regulated proteins .E. Western blotting analysis. F. a.the up-regulated KEGG enrichment bubble. b. The histogram diagram. G. a. the down-regulated KEGG enrichment bubble..b. The histogram diagram.

**Fig.S7**


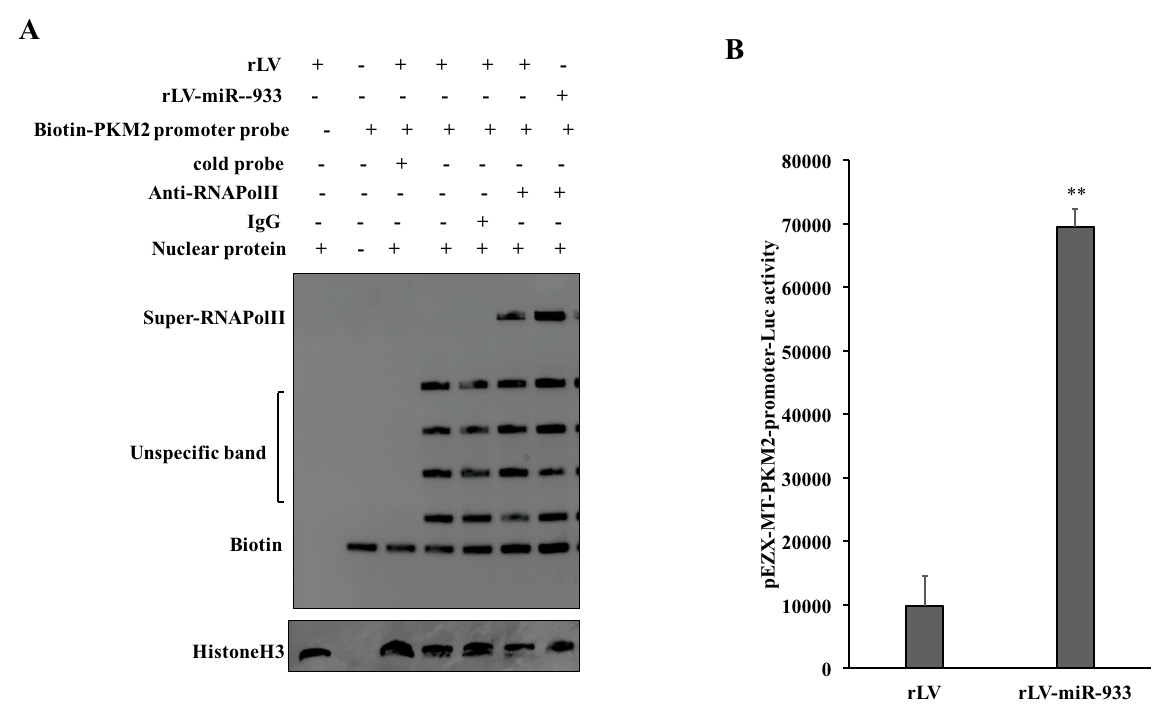


**FigureS7** miR-933 enhances the expression of PKM2 and the interaction between PKM2 and CARM1. A. anti-RNAPolII super-EMSA. B. PKM2 promoter luciferase activity analysis. PKM2 promoter sequence. gattcgctggatttgtctct gccagaggctcgtggacagcatttctagaactgtcagatcaaaatctgcaactgaggagttagtttgccatcaactctacatgtacatttcagtcttgtgaacatatctagatttagaactttttcgtttgtttgtttttgagacaaggtctggctctgtcacggaggctggagtgcagtggagtgatctctgctcactacaacctccacctctcggattcaagcgattctgcctcagcttcccgagtagctgggattacagacgtgcaccaccatgcccagctagtttttgtatttttagtaaagacggggtttcgccacgttggccaggatggtctcaactcctggcctcatgtgattcgccggcctcggcttcccaaagtgttgggattacaggcgtgagccactgcacccagacaatttagaactttaaaatacaatatagtccagcatcggccagatccttgccatcccagtgttgtccaccactgttcttagtttgtcctcagctcaggggccagggagggacttttatgccatccctctttctctgatagagaggaatctaaccctccctaggggggcatgtcttttttccttctcaacaacttcatgttctttcaccaccttaataatcaagcatttccttcccaacaacaactaaagggaccaggaaagacttacagaagaaagtgaaagatttttatctcacttcaaacagaaatttacttttaggctgtcactgtctcctcccagcttatctgagctaacaggaagcgaatgaaggcctgtgattcagctgcaggcagcgggtatgcctgagcaggtcacacccatttggtctcttttgccaggaaaataaaaaaagaacctaagagaacggtcttcacattttgaatgcgcaacattg tatctgtgaatgaaggcaagagttaacagctgtttaattgataactgctc gcatcattagttgctggctaacaactgggaaatcagaaaa

**Fig.S8**


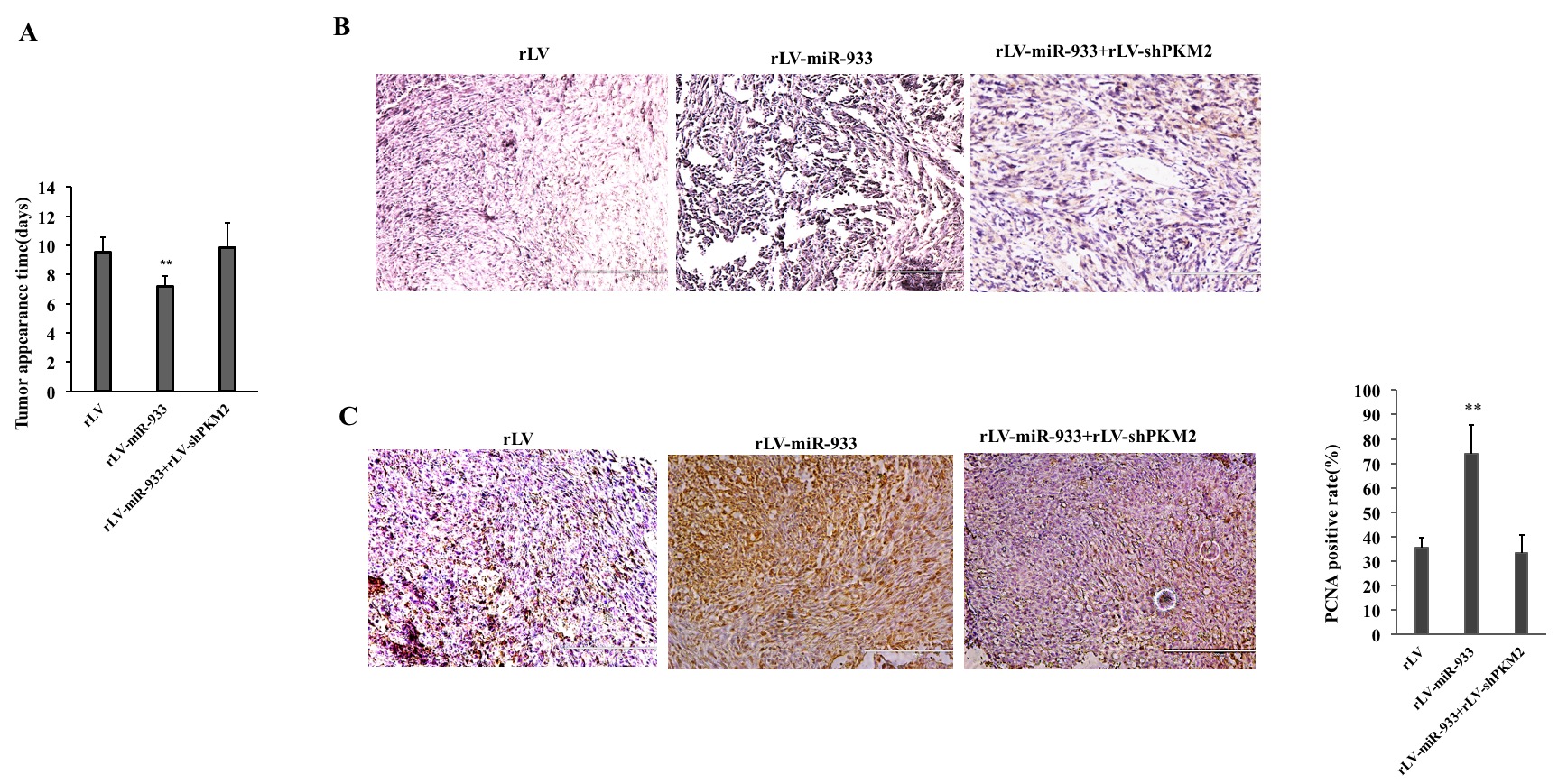


**FigureS8** miR-933 enhances the carcinogenic function dependent on PKM2. rLV-shPKM2 was prepared with pLVX-shRNA-PKM2-tdTomato-Puro.PKM2 shRNA(I)：5’-GTTGCTGTGGCTCTAGACACT-3’and PKM2 shRNA(II) :5‘-cacgtgcccccatcattgctgt-3’.A. the xenograft tumor was dissected. Comparison of tumor appearance time(days). B. The hematoxylin eosin (HE) staining (original registration×100). C. anti-PCNA immunohistochemical staining (original registration×100).
